# Recent advances of CREKA peptide-based nanoplatforms in biomedical applications

**DOI:** 10.1186/s12951-023-01827-0

**Published:** 2023-03-03

**Authors:** Nannan Zhang, Bin Ru, Jiaqi Hu, Langhai Xu, Quan Wan, Wenlong Liu, WenJun Cai, Tingli Zhu, Zhongwei Ji, Ran Guo, Lin Zhang, Shun Li, Xiangmin Tong

**Affiliations:** 1 College of Life Sciences and Medicine, Zhejiang Sci-Tech University, Hangzhou, 310018 China; 2 Laboratory Medicine Center, Clinical Research Institute, Zhejiang Provincial People’s Hospital, Affiliated People’s Hospital, Hangzhou Medical College, Hangzhou, 310014 Zhejiang China; 3 Center for Rehabilitation Medicine, Department of Pain Management, Zhejiang Provincial People’s Hospital, Affiliated People’s Hospital, Hangzhou Medical College, Hangzhou, 310014 Zhejiang China

**Keywords:** CREKA peptide, Active targeting, Fibrin-fibronectin complexes, Nanoplatform, Biomedical applications

## Abstract

Nanomedicine technology is a rapidly developing field of research and application that uses nanoparticles as a platform to facilitate the diagnosis and treatment of diseases. Nanoparticles loaded with drugs and imaging contrast agents have already been used in clinically, but they are essentially passive delivery carriers. To make nanoparticles smarter, an important function is the ability to actively locate target tissues. It enables nanoparticles to accumulate in target tissues at higher concentrations, thereby improving therapeutic efficacy and reducing side effects. Among the different ligands, the CREKA peptide (Cys-Arg-Glu-Lys-Ala) is a desirable targeting ligand and has a good targeting ability for overexpressed fibrin in different models, such as cancers, myocardial ischemia-reperfusion, and atherosclerosis. In this review, the characteristic of the CREKA peptide and the latest reports regarding the application of CREKA-based nanoplatforms in different biological tissues are described. In addition, the existing problems and future application perspectives of CREKA-based nanoplatforms are also addressed.

## Introduction

Nanomedicine is an emerging field that uses nanoparticles to facilitate the diagnosis and therapy of different diseases [1, 2]. The rapid development of nanotechnology and nanomedicine provides numerous nanoplatforms for accuracy and efficient diagnosis and treatment and has great potential for clinical application [1–4]. Therapeutic drugs or imaging probes can be loaded with nanomaterials to increase the stability and metabolic kinetics of drugs in the body, thereby improving the therapeutic or imaging effect on diseases while reducing toxicity and side effects [5–7]. However, the therapeutic of commonly used nano-based drug delivery systems is limited because these nanoplatforms rely only on the enhanced permeability and retention (ERP) effect [8–10]. The active targeting strategy improves the delivery efficiency of nanomedicine to target organs and tissues [11–14]. The targeted drug delivery system was prepared by modifying a specific ligand on the surface of the nanoplatform, which can specifically recognize and bind to overexpressed receptors in tissues or cells to achieve a greater distribution of drugs at target sites, thereby improving the efficacy of the drug and reducing toxic side effects. Currently, commonly used targeted ligands include carbohydrates or polysaccharides [15, 16], peptides [17–22], monoclonal antibodies [23, 24], aptamers [25, 26], and protein ligands [27, 28].

Compared with other targeting ligands, polypeptides have properties similar to antibodies in functional theory and have good biological activities, such as low molecular weight, strong penetrating power, low cost, and lack of immunity, which have attracted increasing research attention. Phage-display technology is a simple and effective peptide screening method that can screen bioactive peptides that specifically bind to disease targets. The polypeptide CREKA (Cys-Arg-Glu-Lys-Ala) was discovered by in vivo screening of phage-displayed peptide libraries for tumor homing in tumor-bearing MMTV-PyMT (mouse mammary tumor virus-polyomavirus middle-T antigen (MMTV-PyMT) transgenic breast cancer mice [29]. After intravenous injection of synthetic CREKA, the peptide was detected in human tumors; but not in normal tissues. In vivo experiments have shown that this tumor-homing pentapeptide binds to clotted plasma proteins, thereby establishing its behavior as a clot-binding peptide [30]. Fibrin clots are formed by the large precursor protein fibrinogen and is present not only in blood clotting but also in almost all forms of tissue injury to initiate hemostasis and acts as a temporary extracellular matrix [31]. In the process of tissue regeneration, repair, and reconstruction, the extracellular matrix provides the necessary environment for the activities of the cell (proliferation, differentiation and migration, etc.). In the case of normal tissue trauma, fibrin is rapidly formed and deposited at the site of injury, as a temporary extracellular matrix, as a "reservoir" of a variety of active substances. In addition, studies on tumor interstitial composition have shown that fibrin is one of the main protein components of the extracellular matrix of tumors [32, 33], and fibrin formation in the tumor stroma is associated with increased tumor vascular permeability and abnormal coagulation function [34]. During tumor progression, a large number of deposited fibrin and fibronectin form fibrin–fibronectin complexes, which provide a more favorable microenvironment for tumor invasion, angiogenesis and distant metastasis [35]. The fibrin-fibronectin complexes within the microthrombi are present on the blood vessels in different models, including cancers [36–38], micro-thrombosis in myocardial ischemia-reperfusion [39], fibrosis [40], and atherosclerosis [41], and are largely undetectable in the circulating blood of normal tissues; therefore, fibrin is undoubted an ideal target for the diagnosis and treatment of many diseases. Unlike fibrin-specific antibodies, CREKA is a less immunogenic, low-cost fibrin-binding peptide and exhibits high affinity for fibrin-fibronectin complexes, so CREKA is considered a promising targeted peptide and can be used in a variety of disease models.

Considering the wide range of the applications of CREKA-based drug delivery systems, there has been no review of their application in the biomedical field. Notably, in this review, current research regarding CREKA peptide-based nanoplatforms and applications in different disease models is summarized. Firstly, the properties of the CREKA peptide and CREKA-based nanoplatforms are elaborated. Second, the application of various CREKA-based nanoplatforms in different tissues, including cancers, thrombosis, and atherosclerosis, is discussed. Finally, the current challenges and future prospects of CREKA-based nanoplatforms in biomedicine are addressed.

## The physical and chemical properties of CREKA pentapeptide

CREKA is a linear polypeptide composed of 5 amino acids arranged by Cys-Arg-Glu-Lys-Ala, obtained by phage display technology, with a neutral charge, which can recognize fibrin-fibronectin complexes formed by coagulated plasma proteins. Its target is the network structure of plasma protein clots, originally discovered by Dmitri Simberg in 2007 [42]. Previous studies have found that the CREKA polypeptide can specifically target fibrin-fibronectin complexes overexpressed in various diseases, including metastatic cancer, renal fibrosis, and thrombosis. The structure of the CREKA polypeptide makes it an attractive polypeptide for nanoparticle targeting because unlike other clot-binding polypeptides, which are cyclic 10 amino acid peptides [33], CREKA is linear and contains only 5 aa. Furthermore, the sulfhydryl groups of individual cysteine residues are not required for binding activity and can be used to couple the CREKA peptide to other moieties. For example, CREKA was successfully modified on the surface of nanoparticles by a Michael addition reaction between the thiol group of cysteine residues in CREKA polypeptides and the maleimide group in nanocarriers, and the binding efficiency of CREKA was above 90% [36, 43, 44]. The in vitro and in vitro results suggest that CREKA-conjugated vectors significantly improve retention and therapeutic outcomes compared to non-targeted controls without CREKA modification [45–47]. In principle, CREKA peptide-modified nanoplatforms can achieve a specific distribution of the loaded drugs at the target position and improve treatment efficiency.

## Applications of CREKA-based nanoplatforms in biomedicine

### Cancer imaging

**Fig. 1:**
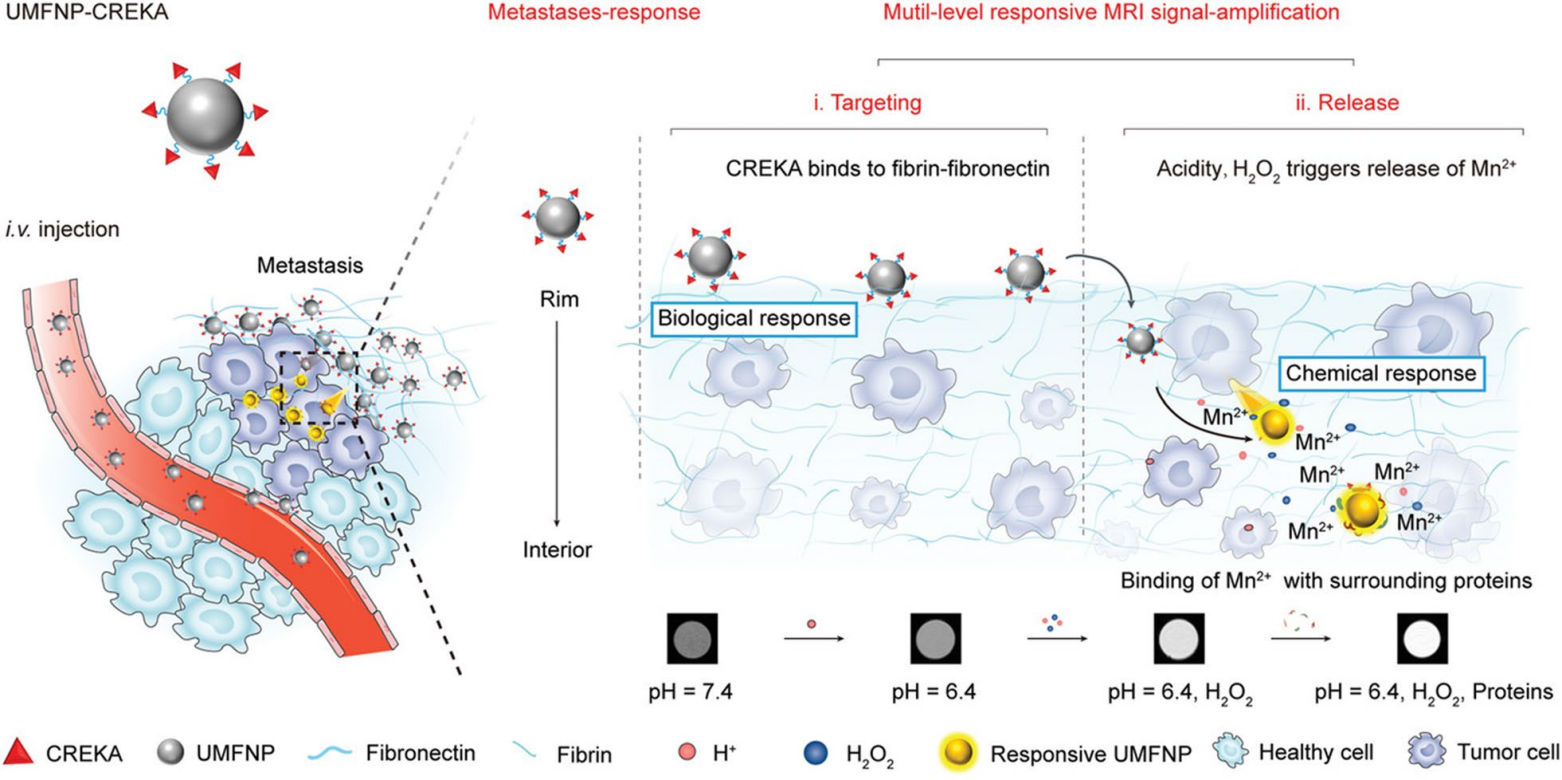
Scheme illustration of the prepared UMFNP-CREKA nanoprobe for imaging ultrasmall metastases by T1-weighted MRI with multiple levels of response [58]

**Fig. 2:**
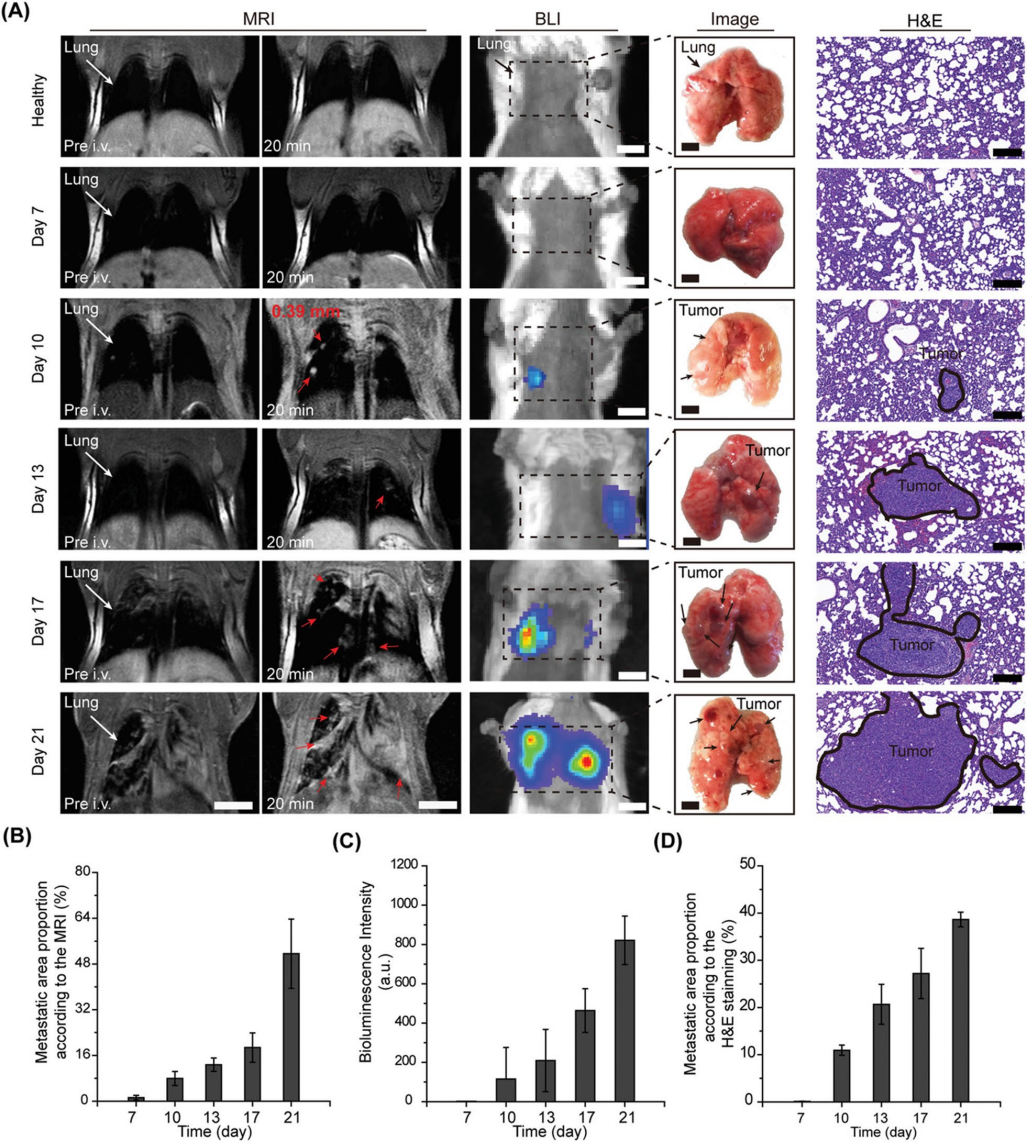
T1-weighted MR imaging of lung metastases in vivo. **A** MR images and H&E stained images after 20 min of injection of UMFNP-CREKA. **B** MRI-based proportion of lung metastases area. **C** Quantified bioluminescence imaging intensity. **D** Proportion of lung metastases area based on H&E staining [58]

Cancer is the main cause of death worldwide, and metastasis is the leading cause of cancer-related death [48]. Therefore, it is important to achieve noninvasive monitoring of tumor exacerbation and metastasis in the early stages for timely intervention and treatment to improve the prognosis of cancer patients [49]. In recent years, molecular imaging technology has made many advances in the imaging diagnosis of a variety of diseases and real-time monitoring of treatment effects [50–57]. Li et al. reported an ultrasensitive T1 weighted magnetic resonance imaging (MRI) nanoprobe UMFNP-CREKA for early diagnosis of ultra-small metastases (Fig. 1) [58]. The nanoplatform was prepared by modifying ultrasmall manganese ferrite nanoparticles (UMFNPs) with the tumor-targeting peptide CREKA to achieve accurate tracking of ultrasmall metastases of breast cancer. In this study, CREKA-conjugated UMFNPs (UMFNP-CREKA) were successfully developed as a novel T1-weighted MRI contrast agent, which was uniform with ultra-small size of 5.6 and 5.7 nm. The in vitro T1 weighted MRI measurements were also conducted to assess the imaging ability of UMFNP-CREKA, and the results showed that the T1 weighted MRI images of UMFNP-CREKA were gradually brightened with the increasing drug concentration, and the r1 relaxivity of UMFNP-CREKA was 6.79 mM^−1^ s^−1^, indicating its potential as a high-quality MRI contrast agent. An in vivo tumor targeting study showed that UMFNP-CREKA-Cy5.5 accumulated more at the tumor site than free Cy5.5 and CREKA-Cy5.5. The fluorescent-positive spots of the UMFNP-CREKA-Cy5.5 group were 6.8-fold higher than those of the CREKA-Cy5.5 group. These above results suggest that CREKA modified preparations could provide tumor-targeting performance, and the targeting efficiency of the nanoprobes was significantly higher than that of small-molecule contrast agent. In vivo MR imaging study, the largest contrast-to-noise ratios (CNR) in the rim and interior of the tumor in the UMFNP-CREKA group was nearly 2.8 and 1.6-fold higher than those in the non-targeting group. Under tumor environment stimulation, UMFNP-CREKA responsively released Mn^2+^ from UMFNPs, and the localized released Mn^2+^ reacted with proteins to amplified T1 weighted signals. Unlike previously reported ferrite nanoprobes based on the EPR effect to improve detection sensitivity, UMFNP-CREKA can detect metastatic tumors as small as 0.39 mm (Fig. 2). The molecular imaging strategy based on the unique features of the cancer/metastasis microenvironment and the physicochemical properties of nanoprobes can improve the detection sensitivity of ultrasmall breast cancer metastasis and enable early diagnosis of metastatic tumors.

**Fig. 3:**
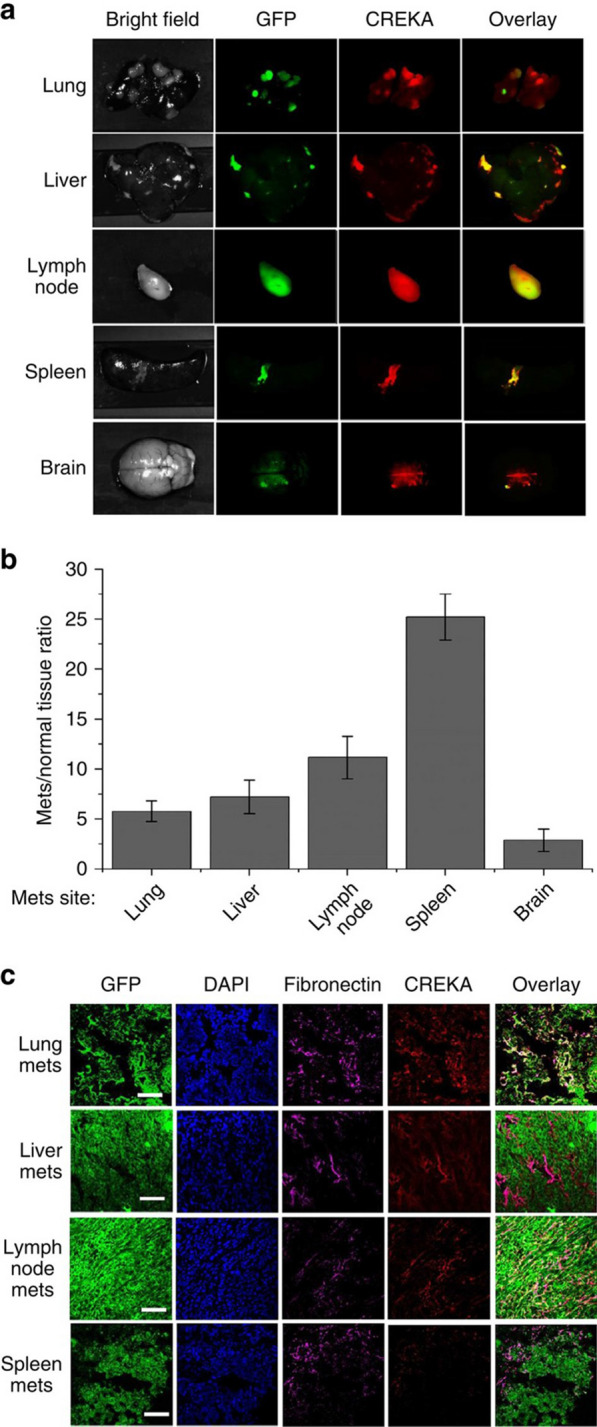
CREKA peptides specifically bind fibronectin-associated complexes in metastatic tumors. **a** Fluorescence imaging of major organ metastatic tissue in mice with spontaneous metastatic 4T1-GFP-Luc2 breast tumors. **b** Fluorescence intensity ratio between metastatic tumor and normal tissue. **c** Fibronectin staining of cryosections of metastatic tumors in different organs as shown in a [37]

Breast cancer is a highly metastatic malignancy, and 1 in 3 breast cancer will develop metastases of distal tissue, resulting in an increased risk of death [59]. Early accurate tracking and differential diagnosis of breast cancer with metastatic potential and micrometastases (< 2 mm) may facilitate the implementation of more effective and time-sensitive treatments and then improve breast cancer treatment efficacy [60, 61]. However, although a variety of imaging techniques are currently available in clinics, such as MRI, B-ultrasound, and mammography for breast cancer detection, they cannot accurately detect the growth and location of early metastases. To improve the sensitivity of MRI for the diagnosis of cancer, Zhou et al. developed a CREKA-targeted MRI nanoprobe, CREKA-Tris (Gd-DOTA)_3_ [37]. The CREKA peptide can specifically bind to the fibrin-fibronectin complex upregulated in the extracellular matrix (ECM) of tumor tissue, thereby avoiding the distribution of MRI contrast agents in normal organs and tissues to improve the detection of micrometastases. The researchers tested the molecular MRI performance of CREKA-Tris (Gd-DOTA)_3_ in a mouse model of metastatic breast cancer. The results indicated that CREKA-Tris (Gd-DOTA)_3_ could bind to fibrin-fibronectin complexes in almost all organ metastases (Fig. 3). In addition, the results showed that CREKA-Tris (Gd-DOTA)_3_ produced strong MRI signaling enhancement in metastatic tumors and was able to detect micrometastases as small as 0.5 mm, increasing the detection limit of current clinical imaging modalities. Furthermore, the researchers also tested the contrast-enhancing effect of the non-targeted contrast agent CERAK (Cys-Glu-Arg-Ala-Lys)-Tris (Gd-DOTA)_3_ in the same spontaneous mouse model and found that only large metastases could be detected using this non-targeted MRI contrast agent. These results suggest the molecular MRI using CREKA-Tris (Gd-DOTA)_3_ as a contrast agent may contribute to the early detection of clinically high-risk breast cancer and micrometastases.

### Thrombi imaging

**Fig. 4:**
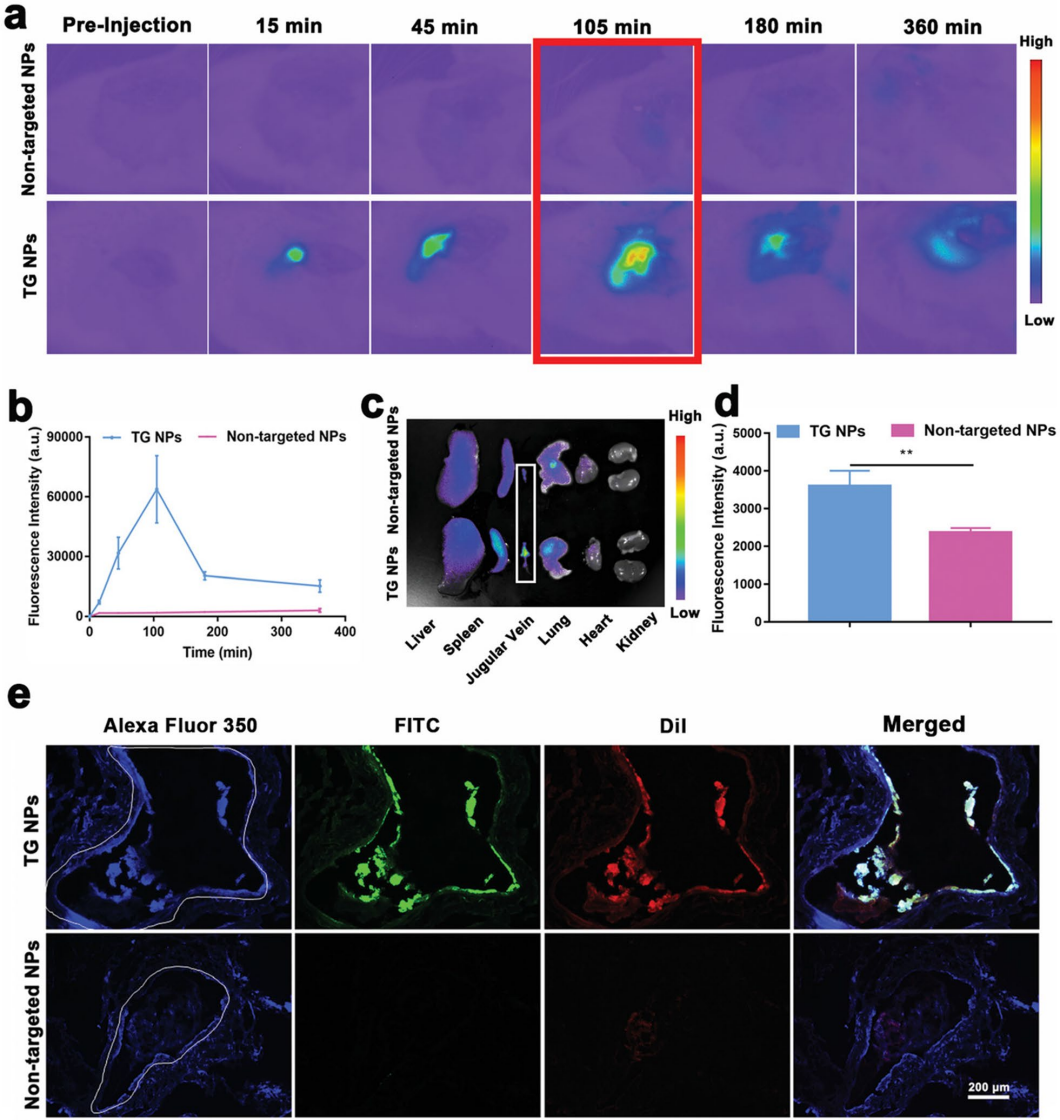
Biodistribution and targeting performance of TG NPs. **a** In vivo fluorescence images in jugular venous thrombosis model rats treated with TG NPs or non-targeted NPs. **b** In vivo thrombosis fluorescence intensity at the corresponding time point. **c** In vivo fluorescence image of major organ after 6 h intravenous injection. **d** Ex vivo fluorescence intensity of thrombosis 6 h after intravenous administration of TG NPs or non-targeted NPs. **e** Fluorescence images of thrombus sections after treatment with TG NPs or non-targeted NPs [64]

Venous thromboembolism (VTE) including pulmonary and deep vein thrombosis (DVT), is a widespread public health problem [62]. As one of the most common and deadly diseases worldwide, VTE is the leading cause of cardiovascular emergencies and deaths [63]. Before treatment, the detection of the precise localization of thrombi is essential. In Liang xingjie’s paper Zhong et al. published in Advanced Materials in 2022 [64], a novel fibrin-specific nanoprobe (NP) was reported for thrombus-specific MRI and photoacoustic (PA) bimodal imaging and prevent thrombosis formation. The probe used poly (lactic acid-co-glycolic acid) as the drug carrier, loaded with MRI contrast agent Fe_3_O_4_, near-infrared (NIR) fluorescent probe (IR 780), and ketotifen (KF), surface-modified CREKA peptide, whose fibrin-specificity distinguishes fibrin-rich thrombs from collagen-rich or erythrocyte-rich thrombi. The targeted NPs (TG NPs) were easily prepared by double emulsion and carbon diimide methods. High-performance liquid chromatography (HPLC) experiments confirmed that the encapsulation efficiency of CREKA peptide in TG NPs was 64.344% ± 10.523, indicating the successful modification of CREKA. T2-mapping MRI was performed in vitro to assess the MR relaxation rate of TG NPs. The results indicated that the MR images of TG NPs with different iron concentrations tended to darken with the increase of concentrations, while the negative signal intensity of TG NPs increased with increasing concentrations. Quantitative analysis showed that the T2 relaxation rate of TG NPs was 79.52 mM^−1^ s^−1^. In addition, the PA signal intensity of TG NPs with different IR780 concentrations increased with the increasing of TG NPs concentration. The above results showed that TG NPs had excellent MRI and PA imaging performance. A jugular venous thrombosis model was established to assess whether TG NPs specifically aggregated in the thrombus. In vivo imaging showed that large amounts of TG NPs accumulated in the thrombus and the fluorescence signal intensity of TG NPs in the jugular vein was significantly greater than that in the non-targeted NPs group (P < 0.01). Notably, the maximum fluorescence emission occurred between 45 and 105 min after intravenous administration, with peaks observed at 105 min before the signal gradually disappeared (Fig. 4). The above results indicated that CREKA coupling improved the targeted delivery ability of TG NPs. Furthermore, in vivo MR/PA imaging of thrombi study showed that after injection of TG NPs, the T2 relaxation rate increased, while the intensity of MR signaling decreased in parts of the thrombosis, especially 3 and 7 days after thrombosis induction, in accordance with the results of T2 mapping. However, this trend was not obvious in the non-targeted NP group. Meanwhile, as a multimodal imaging agent, TG NPs could also be used to detect the PA signal of jugular vein thrombosis. The results indicated that TG NPs could detect thrombus in the left jugular vein, and the PA signal intensity increased significantly 7 days after thrombus induction. The ability of TG NPs to prevent thrombus formation was also investigated in the jugular vein stenosis model. The results showed that TG NPs loading with ketotifen induced mast cell (MCs) suppression that occurred in the venous vessel microenvironment, and the proportion of rats in the TG NPs, ketotifen (KF), polymer compound 48/80, normal saline, and low molecular weight heparin (LMWH) groups showed that thrombosis was 16.67%, 16.67%, 0%, 83.33% and 0%, respectively. In this study, fibrin-specific nanoprobes were designed to identify fibrin-rich thrombus, thereby improving the detection of thrombi and facilitating timely treatment of patients.

**Fig. 5:**
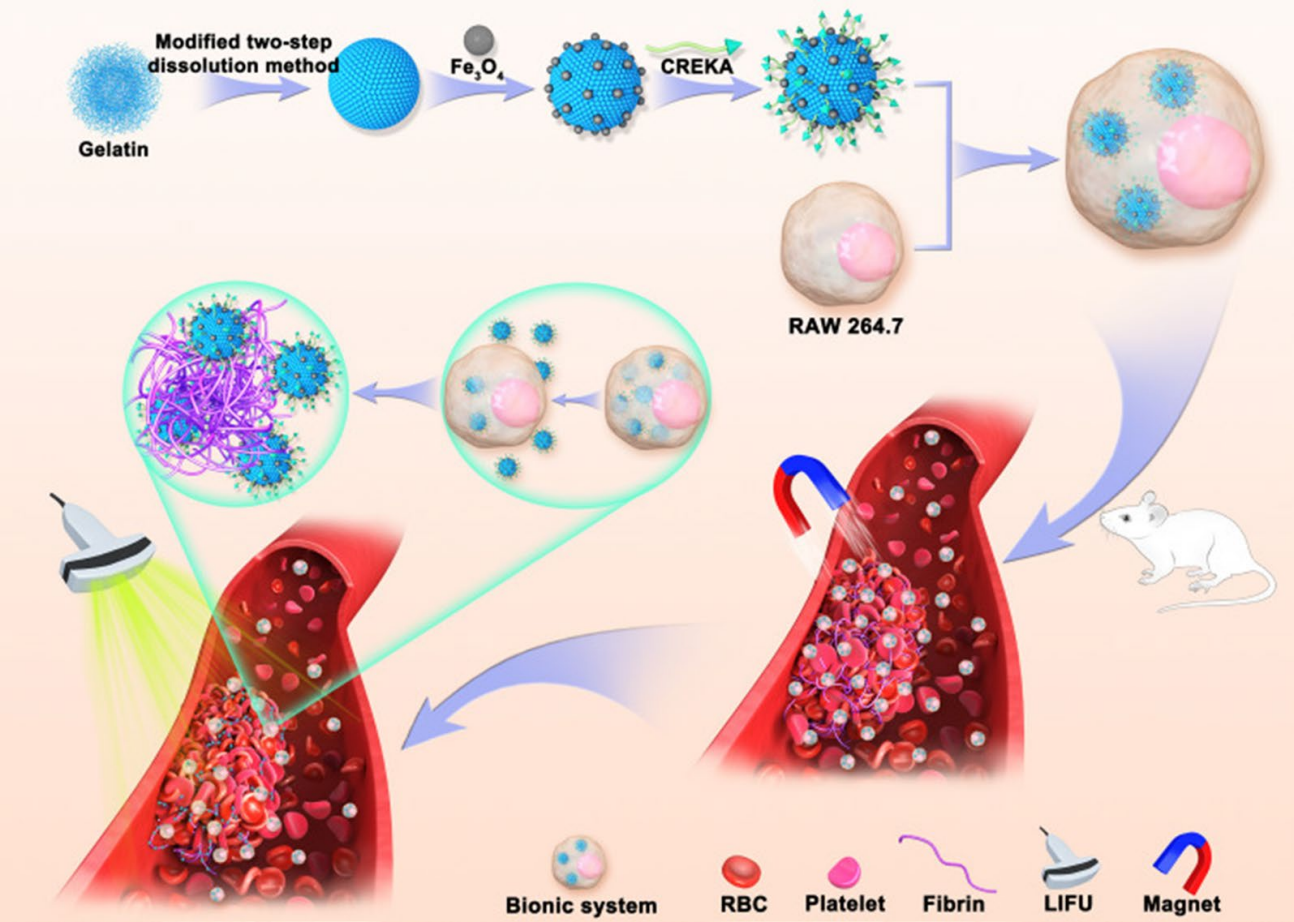
Scheme of the construction of TNPs and their application in targeted imaging of thrombus [65]

Another related research on thrombus imaging using the CREKA peptide-based nanoplatform was published in 2015 by Guo et al. [65]. Arterial thrombosis poses a serious threat to human health. This study designed a specific bionic drug delivery system with enhanced thrombosis-targeting capabilities. This biomimetic nanoplatform used gelatin as the drug carrier, Fe_3_O_4_ as the magnetic navigation medium and MRI contrast agent, modified by CREKA peptide (TNPs), and then phagocytosed by macrophages to form the final formulation (MTNPs, Fig. 5). The results showed that most TNPs were released from macrophages after 10 min of low-intensity focused ultrasound (LIFU) irradiation at 2 W/cm^2^. Fluorescence imaging results showed that the fluorescence intensity of MTNPs + magnet + LIFU treatment group was significantly higher than that in TNPs-treated group at 60 min, 90 min and 120 min, which was attributed to the LIFU irradiation and magnetic navigation. The results of in vivo MR imaging of the bionic system showed no significant signal decrease around the thrombus in the model mice before and after the injection of non-targeted NPs (NTNPs). While the MR signal of the carotid artery decreased in the TNPS treatment group. In addition, the TNPs+magnet treatment group showed a significantly reduced signal around the thrombus compared with the TNPS group. In the MTNPs+magnet+LIFU treatment group, the perithrombotic signal decreased significantly, and the degree of reduction was significantly higher than that in the TNPs and TNPs+magnet groups. This may be attributed to the attraction of MTNP to the thrombus site by the natural platelet-targeting ability of macrophage and magnetic navigation. After LIFU irradiation, MTNPs released TNPs and exposed the CREKA peptide, resulting in NPs continuously targeting fibrin, further enhancing the targeting performance.

### Antithrombotic therapy

Thrombotic diseases caused by thrombosis and thromboembolism are the leading cause of death and disability worldwide [66–69]. Currently, clinically used thrombolytic agents, such as recombinant tissue plasminogen activator (rt-PA) and urokinase (UK), are the most important treatment methods for thrombosis-related diseases [70, 71]. These proteases convert plasminogen into plasmin, which then cleaves the fibrin mesh in the thrombus to achieve thrombolytic therapeutic effects [72]. Unfortunately, these thrombolytic drugs have short circulation times after in vivo administration, non-specific targeting, and serious bleeding complications, which hinder the clinical application of this therapy. Therefore, there is an urgent need for safe and efficient thrombolytic therapies.

**Fig. 6:**
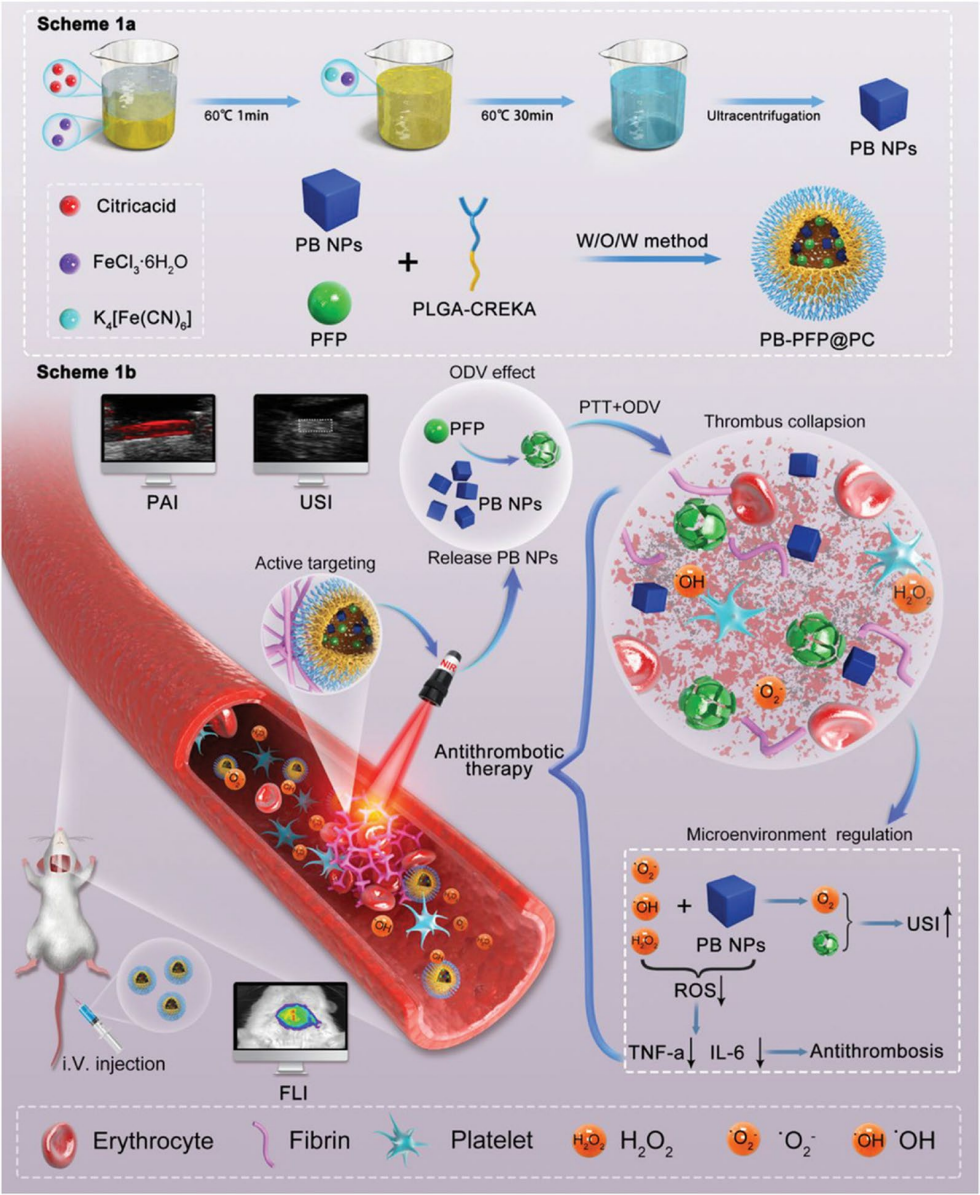
**a** Scheme illustration of the preparation of PB-PFP@PC nanodroplets and **b** its application in specific antithrombotic therapy [73]

Zhang et al. proposed a comprehensive antithrombotic therapy strategy by integrating non-pharmacological methods such as photothermal therapy (PTT), optical droplet vaporization (ODV), and removal of overexpressed ROS in the thrombotic microenvironment to obtain a more effective antithrombotic therapy (Fig. 6) [73]. In this study, thrombo-specific Prussian blue (PB)-based nanodroplets, namely, PB-PFP@PLGA-CREKA (PB-PFP@PC) nanodroplets, were prepared. PB-PFP@PC was prepared using PB and perfluorinated pentane (PFP) in the core, and the targeting peptide CREKA was modified on the PLGA surface. By making full use of the H_2_O_2_ scavenging capacity of PB, an antioxidant reaction could be achieved when PB was released by NIR laser irradiation and H_2_O_2_ stimulation. Enhanced ultrasound imaging (USI) produced by the generated O_2_ enabled real-time monitoring of thrombolytic therapy. The results showed that the prepared PB-PFP@PC had a diameter of 287.58 ± 6.60 nm. During NIR laser irradiation at 1.2 W/cm^2^, the temperature of the PB-PFP@PC increased over time in a concentration-dependent manner. At a concentration of 0.5 mg mL^−1^, the temperature rises to 44.8 °C within 15 min; In addition, PB-PFP@PC released 95% of PB under NIR laser irradiation and H_2_O_2_ exposure, which was due to the H_2_O_2_ sensitivity and photothermal conversion properties of PB-PFP@PC. The results of the targeting ability of PB-PFP@PC on thrombosis indicated that the PB-PFP@PC group had more red fluorescence in blood clots, and exhibited superior targeting efficiency and penetration ability than the non-targeted PB-PFP@P group in the carotid thrombosis model. Finally, the results of in vivo antithrombotic therapy study showed that under the NIR irradiation (1.2 W/cm^2^), the local temperature change of the right carotid artery site in the normal group and the control group increased by approximately 6.8 and 10.2 °C, respectively. The temperature of PB-PFP@P and PB-PFP@PC under NIR irradiation increased by about 16.6 and 18.3 °C, respectively, confirming the effective target accumulation of PB-PFP@PC and the possibility of damaging thrombosis in vivo through local PTT and ODV effects. To evaluate the thrombolysis effect of PB-PFP@PC, SD rats with carotid artery thrombosis were used as animal models, and the therapy level was calculated in accordance with the following formula: Involving range % = destroyed area/total vascular area × 100%. By observing the destruction range of thrombolytic effect after 1 day of in vivo administration, it was found that compared with the control group, PB@P group (20.7%), PB-PFP@P group (45.7%), and PB-PFP@PC group (59.32%) had obvious thrombosis rupture, of which the PB-PFP@PC group achieved better thrombotic destruction effect due to the combination of PTT, ODV effect, and fibrin specific targeting. After 7 days of the administration, the treatment efficacy results showed that the PB@P (27.6%), PB-PFP@P (54.5%), and PB-PFP@PC (74.4%) groups maintained a significant thrombolytic effect, and no thrombosis was observed to reform. In addition, ROS, TNF-α, and IL-6 expression in thrombotic tissues were significantly reduced in the PB@P, PB-PFP@P, and PB-PFP@PC groups after 7 days of treatment. It is worth noting that the expression of TNF-α and IL-6 in the PB-PFP@ PC group was significantly lower than that in the PB-PFP@P group, indicating that the effect of CREKA targeting fibrin could increase the accumulation of PB-PFP@PC in thrombotic tissues and help exert more powerful anti-inflammatory activity.

**Fig. 7:**
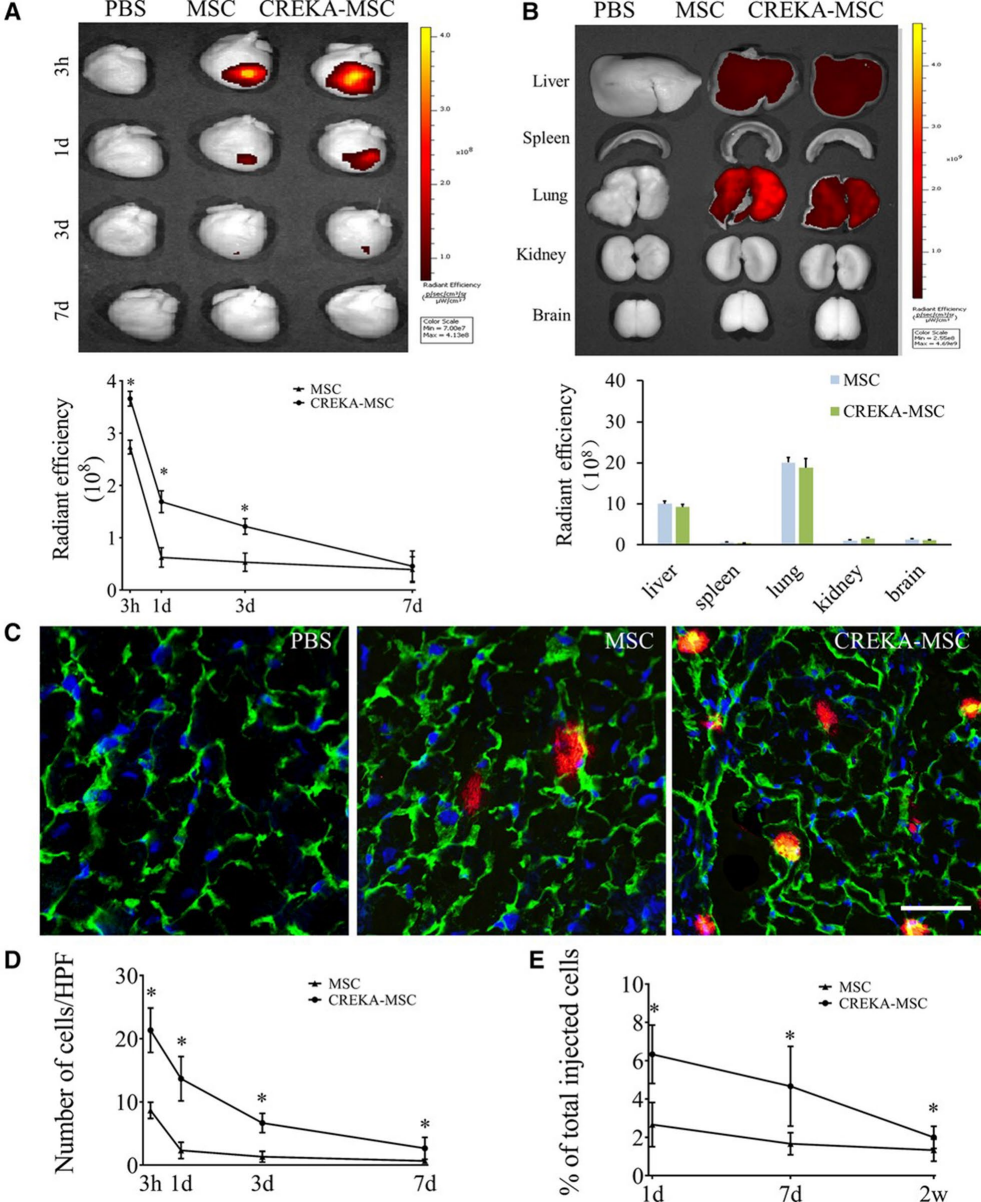
Distribution of CREKA-MSCs in the area of cardiac infarction. **A** Ex vivo optical imaging and semi-quantitative results of the heart injected with DiD-labeled CREKA-MSCs at different time points. **B** Ex vivo optical imaging and semi-quantitative analysis of other major organs in model mice treated with DiD-labeled CREKA-MSCs 3 h. **C** Fluorescence micrographs of the distribution of CREKA-MSCs in the infarct area. **D** Quantitative analysis of transplanted cells. **E** Quantitative PCR of male-specific Sex-determining Region Y gene (SRY) at 1 day, 7 days and 2w after cell infusion [74]

In another study, a stem cell-CREKA-fibrin targeting system was developed for the treatment of myocardial ischemia-reperfusion (MI/R) injury in rats [74]. Previous studies have found that poor cell homing is a major barrier limiting the efficacy of cardiac cellular therapy. Overexpression of fibrin after myocardial injury is an ideal target for efficient cell delivery. In this study, the nanoplatform was prepared by first attaching the homing peptide CREKA to liposomes (CREKA-Lipos) and then using lipid membrane fusion technology and lipid bilayer fluidity, fused with stem cells (such as mesenchymal stem cells (MSCs)) membranes. In this study, the real-time expression of fibrin in the MI/R rat model was examined, and it was found that the fibrin expression increased at the onset of myocardial injury and peaked at 24 h of molding, and then significantly decreased and almost disappeared at 7–14 days after molding. Specific expression of fibrin in the myocardial injury region of the MI/R model was demonstrated, providing a basis for CREKA peptide-mediated targeted cell delivery. The in vitro fibrin binding of CREKA-MSCs showed that the number of bound CREKA-MSCs was 2.6- and 2.3-fold higher than that of non-targeted MSCs group under static and flow conditions, respectively. The in vivo distribution results showed that the cardiac fluorescence intensity of the CREKA-MSCs group peaked at 3 h and then decreased slowly. Compared to the MSCs group, the CREKA-MSCs group had significantly stronger fluorescence at different time points (3 h, 1 day, 3 days) after administration (P < 0.05). However, there was no significant difference between the two groups at 7 days. After injection for 1 day, the accumulation of CREKA-MSCs in the myocardium of injured rats was 6.5 times higher than that in the non-targeted MSCs group (Fig. 7). Furthermore, cardiac function was examined using echocardiography and histopathology after 4 weeks of treatment. The results showed that the initial left ventricular ejection fraction (LVEF) was similar in all groups, indicating a comparable degree of initial myocardial injury. After 4 weeks of treatment, the CREKA-MSCs-treated group exhibited the highest LVEF, and other echocardiographic parameters, including fraction shortening (FS), left ventricular internal end-diastolic diameter (LVEDd), and left ventricular internal end-systolic diameter (LVED), showed the same trend. These results indicated that CREKA-MSCs had a better therapeutic effect than non-targeted MSCs therapy, indicating that CREKA modification enhanced the distribution of MSCs in myocardial infarction tissue and translated into additional functional benefits of stem cell therapy in MI/R rat models. Therefore, this fibrin-targeting CREKA-modified transplanted cell delivery system provided an effective platform for regenerative medicine.

### Anticancer therapy

**Fig. 8:**
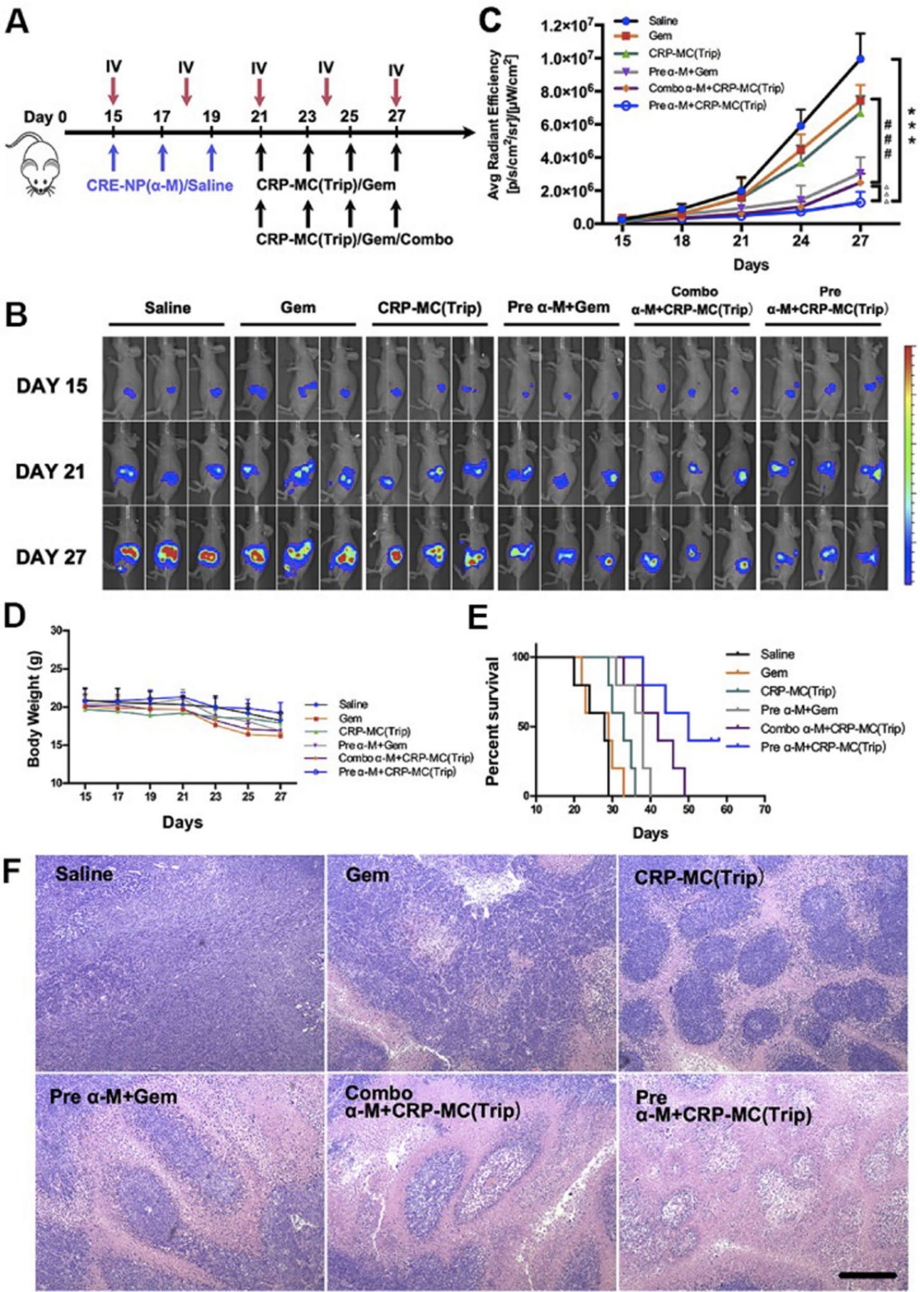
CRE-NP (α-M) pretreatment improved the antitumor effect of CRP-MC (Trip) in pancreatic cancer. **A** Schematic diagram of different treatment modes in situ tumor model. **B** IVIS photographs of mice after different treatments. **C** Tumor growth curves of mice in different treatment groups. **D** Mice weight change curve. **E** Survival ratio of mice in different treatment groups. **F** H&E staining of tumor tissues in different treatment groups [82]

Pancreatic ductal adenocarcinoma (PDAC) is a solid malignancy with a very poor prognosis [75]. The efficacy of conventional therapies has been largely hampered by the dense desmoplastic tumor stroma comprising more than 90% of the total tumor volume, limiting effective drug distribution and penetration [76]. The main cell of PDAC tumor stroma is activated pancreatic stellate cells (PSCs) or cancer-associated fibroblasts (CAFs), which secrete large amounts of extracellular matrix (ECM) to compress blood vessels within the tumor and hinder the efficient delivery of chemotherapy drugs [77, 78]. TGF-β plays an integral role in promoting CAFs activation and tumor angiogenesis [79]. After activation by TGF-β, resident fibroblasts are converted to CAFs, secrete abundant ECM proteins [80], and induce the expression of fibrotic genes such as fibronectin, proteoglycans, laminin, and collagen. In addition, TGF-β enhances tumor angiogenesis by promoting pericyte-endothelial interactions and coverage, reducing lumen size, and vascular access [81]. Thus, down-regulation of the TGF-β signaling pathway inhibits CAFs activation, thereby lowering the tumor stromal barrier and then enhancing the delivery of chemotherapy drugs. Feng et al. developed CAFs-targeted biodegradable polymer nanoparticles (CRE-NP (α-M), modified with CREKA peptide and loaded with traditional Chinese medicine (TCM) α-mangostin (α-M), which efficiently delivered α-M to tumor tissues by CREKA specific recognition of overexpressed fibronectin on CAFs, and then modulated the tumor microenvironment by interfering with the TGF-β/SMAD signaling pathway by α-M. Meanwhile, another cancer cell targeting peptide CRPPR-modified low pH-triggered micelles was prepared and another TCM triptolide (Trip) was loaded to increase the therapeutic effect of Trip at the tumor site and reduce its damage to major organs [82]. The results showed that the average size of CRE-NP (α-M) was 106.93 nm, and the drug loading was 49.62%, with controlled and sustained α-M release. In vitro cell studies showed that the cellular uptake of CRE-NP in TGF-β activated NIH3T3 cells was 1.94-fold higher than that of unmodified NP at 3 h. While there was no significant difference in cellular uptake between CRE-NP and non-targeted NP incubated with non-activated NIH3T3 cells, this may be due to the specificity between CREKA peptide and highly overexpressed fibronectin in TGF-β activated NIH3T3 cells. Biodistribution studies showed that the PANC-1/NIH3T3 mouse model treated with CRE-NP showed a much stronger fluorescence signal in the tumor region compared with non-modified NP. The effect of CRE-NP (α-M) on the regulation of the tumor microenvironment in vivo was investigated. Immunohistochemical staining of the tumor sections showed that both NP (α-M) and CRE-NP (a-M) reduced fibronectin expression in a dose-dependent manner. In addition, Masson’s trichrome test showed that the collagen in the tumor was significantly reduced after the third injection of NP (α-M) and CRE-NP (α-M) compared with the saline group, and the CRE-NP (α-M) group showed the highest degree of collagen reduction. Furthermore, DiR-labeled CRP-MC was used to determine whether modulating the tumor microenvironment with CRE-NP (α-M) could enhance the delivery and distribution of CRPPR-modified micelles in the tumor region. In vivo imaging data showed that the tumor fluorescence signal of the unmodified micelle group gradually increased over time, and CRPPR-modified micelles exhibited stronger tumor accumulation compared to unmodified micelles. It is worth noting that even at 24 h post-dose, CRE-NP(α-M)-treated mice showed the strongest fluorescence signal and longest retention time of CRPPR-modified micelles in the tumor region, confirming that CRE-NP(α-M) improved tumor stromal barriers through CAFs and collagen downregulation, enhancing the distribution of CRP-MC in tumors. To assess the therapeutic advantages achieved of the above therapeutic strategies against stroma-rich tumor models, PANC-1 cells were inoculated in situ into the pancreas of mice. The investigators evaluated the therapeutic efficacy of a combination of CRE-NP(α-M) and CRP-MC(Trip). The results showed that gemcitabine after CRE-NP (α-M) administration was a strong tumor suppressor treatment, while the tumor volume increased rapidly in mice injected with gemcitabine and saline alone (P < 0.05) (Fig. 8). However, the mice were poorly tolerated in the gemcitabine group and CRE-NP (α-M) + gemcitabine combination treatment group due to 19.27% and 15.9% weight loss, respectively. CRP-MC (Trip) had a tumor-suppressing effect similar to that of the gemcitabine. CRP-MC (Trip) and CRE-NP (α-M) pretreatment or simultaneous administration significantly inhibited tumor growth, and CRP-MC (Trip) and CRE-NP (α-M) pretreatment achieved the strongest anti-tumor effect and completely controlled tumor progression. H&E staining of tumor tissues also showed that CRP-MC (Trip) and CRE-NP (α-M) pretreated tumor cells had the most obvious apoptosis and necrosis, whereas the CRP-MC (Trip) group had no cytotoxic effect on central tumor cells. The above studies showed that this sequential targeted delivery nanoformulation provides a new option for PDAC treatment by improving the tumor microenvironment to overcome drug penetration barriers and improve therapeutic efficacy.

However, because breast cancer cells are prone to metastases, the clinical efficacy of commonly used chemotherapeutic drugs for invasive breast cancer is often unsatisfactory. Therefore, it is necessary to simultaneously improve the efficacy of chemotherapeutic drugs on primary tumors and metastases. Fibronectin and its complexes are also overexpressed in tumor vessel walls and tumor stroma. Moreover, the expression of fibronectin in metastatic lesions is even higher than that in primary tumor tissue. Jiang et al. reported CREKA-modified liposome-loaded doxorubicin (CREKA-Lipo-Dox) that targeted fibronectin in ECM for the treatment of metastatic breast cancer [83]. The results showed that the size of CREKA-Lipo-Dox was approximately around 160 nm. This particle size facilitated the passive targeting of CREKA-Lipo-Dox, thereby increasing the accumulation of liposomes in tumor tissue. The results of the in vitro release study showed that CREKA-Lipo-Dox exhibited sustained release characteristics, with no significant burst release compared to free Dox. Cellular uptake experiments revealed that the breast cancer cell 4T1 uptake of CREKA-Lipo-Dox increased nearly fourfold compared to the non-targeted PEG-Lipo-Dox group. The results of in vivo anti-tumor studies showed that the average tumor volume in the PEG-Lipo-Dox and CREKA-Lipo-Dox groups decreased to 150 mm^3^ and 60 mm^3^, and tumor images at the end of treatment also showed significant anti-tumor effects of CREKA-Lipo-Dox treatment. Furthermore, mice treated with CREKA-Lipo-Dox exhibited fewer lung metastatic nodules than other groups. Immunohistochemical staining of tumor sections showed that the fluorescence of CREKA-lipo-DiD co-localized with fibronectin immunofluorescence, whereas free DiD and PEG-lipid-DiD had almost no binding to fibronectin in tumor sections. Anti-CD31 antibody immunofluorescence showed that many blood vessels were formed in tumor tissue. Compared with the non-targeted PEG-lipid-DiD group, CREKA-lipid-DiD could specifically bind to tumor blood vessels. Therefore, the binding of fibronectin to CREKA peptide may be an attractive therapeutic strategy for metastatic breast cancer in the future.

**Fig. 9:**
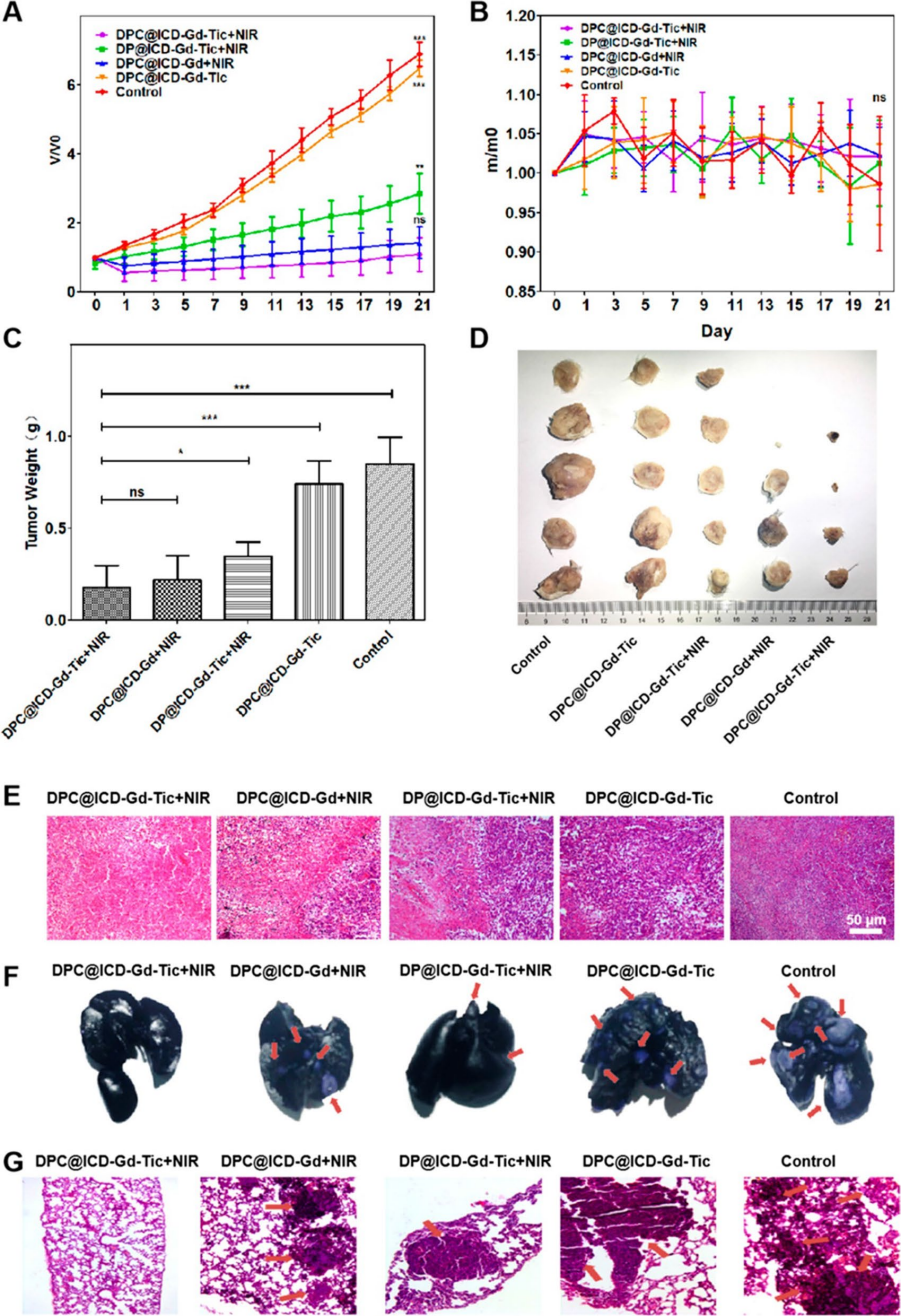
**A** Changes in tumor volume in mice after different treatments. **B** Changes in body weight after different treatments. **C** Tumor body weight at the end of treatment. **D** Tumor images of mice in different groups at the end of treatment. **E** H&E stained sections of tumor tissue. **F** Photographs of the gross morphology of mouse lungs after different treatments. **G** H&E stained sections of mouse lung metastases. Arrows indicated metastases [84]

Zhang et al. proposed an activatable NIR fluorescence/MR bimodal imaging-guided anti-tumor metastasis treatment strategy based on platelet inhibitors combined with photothermal therapy [84]. In this study, the NIR photothermal agent IR780 and the MRI contrast agent Gd-DOTA formed amphiphilic molecules (ICD-Gd) by disulfide bond conjugation, self-assembled with DSPE-PEG-CREKA to form lipid nanoparticles, and loaded with the platelet inhibitor ticagrelor (Tic) to prepare a multifunctional nano-delivery system (DPC@ICD-Gd-Tic). Previous studies have found that the poor prognosis of most malignant tumors is inversely correlated with the number of platelets at the tumor site, and inhibition of platelet function can be an effective way to reduce metastasis. Tic can bind to platelet P2Y12 receptors, thereby reducing the binding of platelets to tumor cells to inhibit metastasis. Based on this strategy, DPC@ICD-Gd-Tic integrated photosensitizers and platelet inhibitors to achieve photothermal therapy in synergistic chemotherapy, thereby ablating the primary tumor and effectively inhibiting its distal metastasis. The fluorescence of IR780 was initially completely masked due to self-aggregation quenching. When DPC@ICD-Gd-Tic was distributed in the tumor site through the active targeting of CREKA and the EPR effect, the disulfide bonds in the system were effectively cleaved by intracellular glutathione (GSH) in the acid tumor microenvironment, resulting in the decomposition of DPC@ICD-Gd-Tic and fluorescence recovery of IR780, in addition, the released Gd-DOTA could achieve T1 contrast-enhanced MRI, thus providing an effective method to monitor the distribution of drugs in the body and imaging of tumors. The TEM results show that the prepared DPC@ICD-Gd-Tic had a monodisperse spherical structure, and the particle size was mainly distributed between 80 and 130 nm. DPC@ICD-Gd-Tic nanoparticles did not fluoresce when excited by 740 nm irradiation, and the fluorescence signal of DPC@ICD-Gd-Tic recovered after incubation with GSH for 8 h. The results of in vivo biodistribution showed that the tumor fluorescence intensity of the DPC@ICD-Gd-Tic group was much stronger than that of the non-targeted DP@ICD-Gd-Tic group, indicating that the modification of CREKA could mediate specific recognition of fibrin-fibronectin complexes in microthrombi to improve efficient drug delivery at tumor sites, whereas DP@ICD-Gd-Tic without CREKA peptide modification relies only on tumor EPR effect. In vivo T1-weighted MRI results showed that the MR signal of the tumor sites in the two groups gradually increased with time, reaching the maximum signal 8 h after injection. The tumor MR signal in the DPC@ICD-Gd-Tic group was significantly higher than that in the non-targeted DP@ICD-Gd-Tic group, which was consistent with the results of fluorescence imaging. The in vivo photothermal therapy and anti-metastasis study showed that, compared with DP@ICD-Gd-Tic + NIR group (4 metastatic foci per lung), only 2.2 foci per lung were observed in the DPC@ICD-Gd-Tic + NIR group, which may be associated with more drug distribution in the tumor site (Fig. 9). The number of lung metastases in 4T1 breast cancer cell tumor-bearing mice in the DPC@ICD-Gd-Tic treatment group was reduced by approximately 90%, the volume of primary tumors was reduced by approximately 70%, and half of the mice were completely cured. Therefore, as a highly specific tumor-homing peptide, CREKA targets fibrin-fibronectin in ECM, which can improve the transport of drugs to tumor neovascularization and achieve better anti-tumor therapeutic effects.

### Other application

Atherosclerosis is one of the main causes of cardiovascular and cerebrovascular diseases, which remained asymptomatic for decades until thrombosis and rupture of arterial plaques, leading to acute coronary syndrome and cardiac arrest [85–87]. MRI is a noninvasive, non-ionizing radiation imaging technique that can be used to assess the extent of atherosclerotic plaques with high spatial resolution and excellent soft tissue contrast. However, the currently clinically used MRI technique lacks sensitivity to preventive medicine, which limits the ability to identify and observe vulnerable plaques. Poon et al. designed hybrid metal-oxide-peptide amphiphile micelles (HMO-Ms) consisting of an inorganic magnetic iron oxide (Fe-Ms) or manganese oxide (Mn-Ms) with the organic, fibrin-targeted peptide CREKA for MR imaging of potential thrombosis on atherosclerotic plaques [88]. The results showed that the prepared amphiphilic self-assembled spherical nanoparticles were 20–30 nm, and the in vitro MR relaxation studies exhibited that HMO-Ms had ultra-high r_2_ value of 457 mM^−1^ s^−1^ and r_1_ value of 0.48 mM^−1^ s^−1^ for Fe-Ms and Mn-Ms, respectively. The MR imaging results of nanoparticles on clots showed that the signal brightness enhancement (T1 increase and T2 decrease) effect of Fe-Ms and Mn-Ms binding clots was statistically significant compared with non-targeted nano preparation. Both Mn-Ms and Fe-Ms could similarly increase the contrast intensity of the MR image: 62% for Mn-Ms and 65% for Fe-Ms, compared to 34% for non-targeted-Mn-Ms and 35% for non-targeted-Fe-Ms, respectively. This imaging technique, based on a novel hybrid MRI nanoprobe, has potential for the specific detection of thrombosis during the pathogenesis of atherosclerosis.

**Fig. 10:**
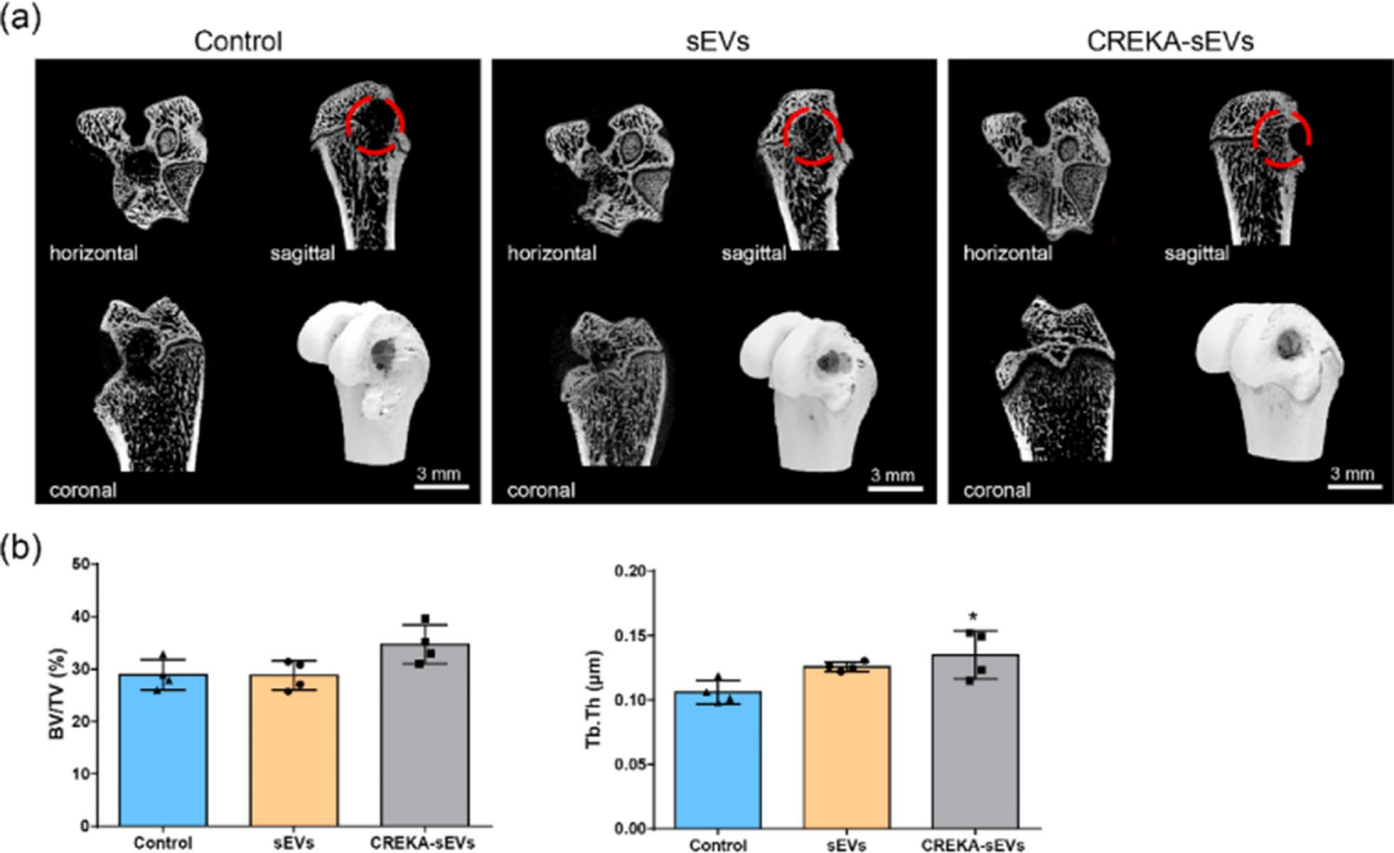
Micro-CT imaging of the femur. **a** Three-dimensional reconstruction and CT images of bone defects in different treatment groups; **b** Quantification of Micro-CT [93]

Bone repair is highly regulated by a large number of bioactive factors [89, 90], and providing a “mixture” containing bioactive factors to the bone defect site may facilitate repair. Previous studies have shown that mesenchymal stem cell-derived small extracellular vesicles (MSC-sEVs) have great potential in tissue repair [91, 92], but the poor homing capacity and in vivo retention ability of sEVs lead to relatively low efficiency of MSC-sEVs treatment, which seriously hinders its application in tissue repair. In order to solve the above problem, Wu et al. developed CREKA-modified sEVs for the treatment of bone repair [93]. Since fibrin is widely expressed in almost all damaged tissues, the fibrin-targeted peptide CREKA was modified on the surface of sEVs, enabling the constructed CREKA-sEVs to acquire the ability to enrich at tissue defect sites. The results showed that the average diameter of CREKA-sEVs was 90.06 nm, and that the modification of CREKA did not affect the inherent biological activity of sEVs. Both sEVs and CREKA-sEVs exhibited biological functions such as promoting osteogenic differentiation of bone marrow mesenchymal stem cells (BMSCs), promoting angiogenesis of endothelial cells and regulating the phenotype of macrophages in vitro. The biodistribution of CREKA-sEVs was investigated in the femoral defect model, and it was found that the fluorescence intensity of the CREKA-sEV group was approximately 1.9-fold than that of the non-targeted sEVs group, indicating that the modification of CREKA could significantly increase the accumulation of sEVs in the bone defect. Micro-CT of the femur sample was performed 4 weeks after surgery. The results showed that the size of bone defects in the control group was almost the same as that in the original group, and trabecular structures appeared loosely in the defects of the sEVs group, while in the CREKA-sEVs group, new bones with dense trabecular structure appeared at the defects, and the defect area was significantly reduced. The results of H&E staining and Masson staining showed that new tissue was filled in both the sEVs group and CREKA-sEVs group, while the control group still had large cavities. In addition, more mature bone tissue with orderly bone trabecular structures and relatively less immature bone tissue were observed in the CREKA-sEVs group than in the non-targted sEVs group (Fig. 10). Thus, this “sEVs-CREKA-fibrin" targeting system enhanced the fibrin binding and retention capacity, significantly enhancing bone defect repair in a rat femoral defect model. This study provides a new strategy for improving the therapeutic efficiency of sEVs and demonstrated that CREKA-sEVs have great potential for application in bone tissue repair.

**Fig. 11:**
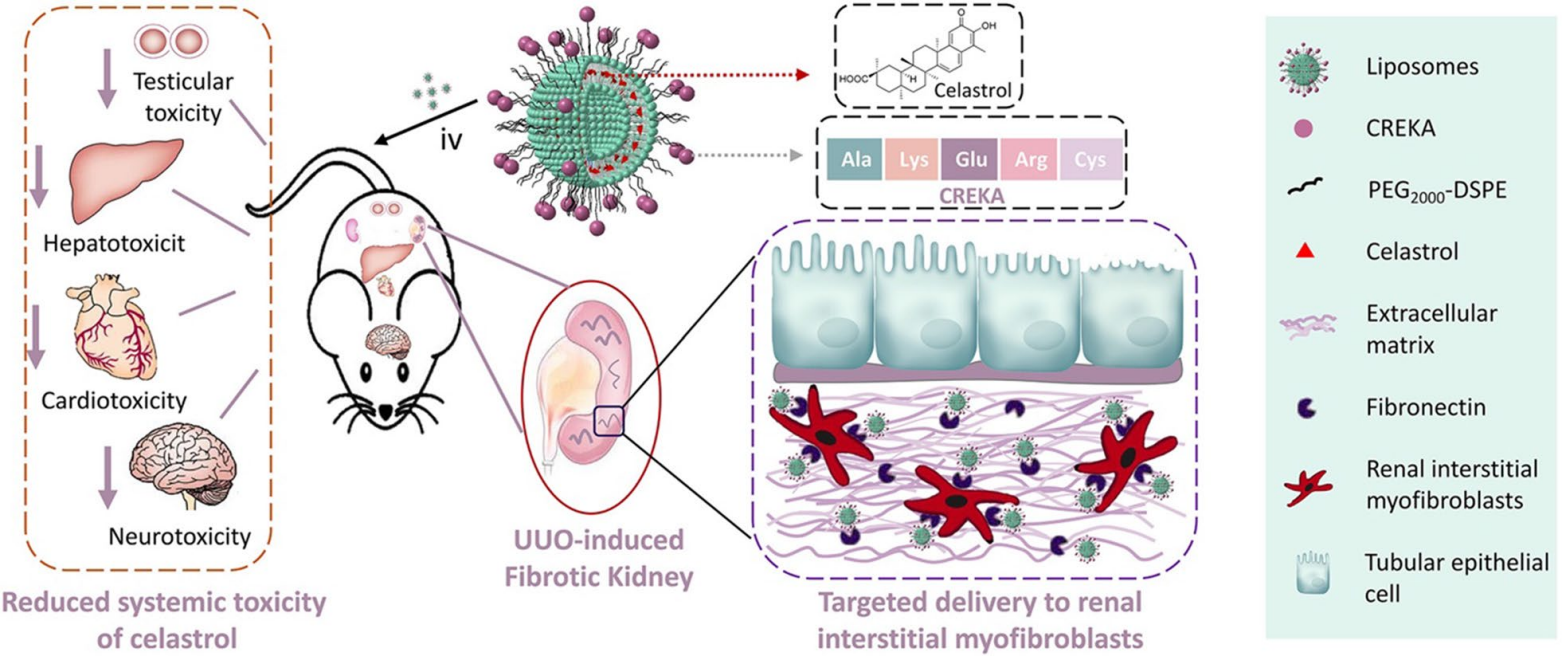
Scheme illustration of the construction of CREKA-Lip/CEL and its targeting delivery of celastrol to renal interstitial myofibroblasts [40]

Chronic kidney disease (CKD) is the progressive and irreversible deterioration of renal excretion [94]. Almost all types of CKDs usually occur in renal fibrosis, leading to the activation of renal interstitial fibroblasts and the accumulation of extracellular matrix [95–97]. These pathological changes lead to end-stage renal disease, which eventually requires dialysis or kidney transplantation [98]. However, currently, there is no treatment that can inhibit or reverse renal fibrosis. Celastrol (CEL) is a pentacyclic triterpenoid in Thunder of god vine (TGV) that can be used to alleviate renal fibrosis caused by mesangioproliferative glomerulonephritis in rats or unilateral ureteral obstruction (UUO) in mice [99, 100]. However, systemic administration of CEL is distributed throughout the body, resulting in severe organ toxicity in the heart, liver, and nervous system [101, 102]. Therefore, specific delivery of CEL to the tissue of the lesion is required to improve efficacy and reduce its side effects. Previous studies have found that myofibroblasts in fibrotic kidney tissue overexpress fibronectin in the extracellular matrix [103, 104]. Therefore, CEL can be targeted for delivery by specifically recognizing fibronectin in fibrotic kidney tissue. The specific binding performance of CREKA peptide has been applied in several studies such as tumor imaging and therapy. However, it remains unclear whether CREKA can be used to target interstitial myofibroblasts for the treatment of renal fibrosis. Li et al. reported a CREKA-modified liposome (CREKA-Lip) nanoplatform for the targeted delivery of CEL for the treatment of renal fibrosis (Fig. 11) [40]. The TEM and DLS results showed that the prepared CEL-loaded CREKA-Lip has a diameter of approximately 110 nm, a small polydispersity index, and good stability. Cellular uptake experiments showed that the drug fluorescence intensity of myofibroblasts treated with CREKA-Lip was significantly higher than that of cells treated with non-targeted Lipo, increasing by 1.8, 2, and 4 times after co-incubation for 1, 2, and 4 h, respectively. The in vivo therapeutic effects of CREKA-Lip/CEL were evaluated on the UOO-induced renal fibrosis model. The results showed that mice treated with CREKA-Lip/CEL had significantly less collagen accumulation, whereas little improvement was detected in mice treated with CEL or non-targeted lip/CEL. Fibrosis genes such as α-SMA, Col1a1, fibronectin, and TIMP-1 in the kidney tissues of mice in the CREKA-Lip/CEL treatment group were decreased at the mRNA level, and the protein levels of Α-SMA, Collal, and fibronectin also showed significant decreased compared to other treatment groups. CREKA-Lip/CEL administration significantly reversed UUO-induced increases in kidney weight and serum urea levels. CREKA-Lip/CEL also greatly inhibited the mRNA expression of inflammatory cytokines, such as TNF-α, IL-1β, ICAM-1, and F4/80. However, treatment with free CEL or Lip/CEL was much less effective. The study showed that targeted drug delivery based on CREKA peptide can be used to target interstitial myofibroblasts for the treatment of renal fibrosis.

## Conclusion and perspective

Nanomedicine is an emerging field that uses nanoplatforms to facilitate disease diagnosis and treatment. Modifying specific target recognition peptides, antibodies, aptamers, etc. on the surface of the nanoplatforms is helpful to improve the distribution of the nano drug delivery system in the lesion tissue, enhance drug efficacy, and reduce toxicity and side effects. Fibrin clots are formed by the large precursor protein fibrinogen after almost all forms of tissue damage. It is involved not only in blood clotting but also in tissue damage and healing, and is highly expressed in thrombosis, tumors, and other damaged tissues. CREKA, a clot-binding pentapeptide, obtained by in vivo phage display, was recently found to recognize fibrin. In previous studies, nanoplatforms based on CREKA have shown excellent specific recognition and binding to fibrin clots, and have been used in the imaging and treatment of a variety of biomedical tissues and disease. In this article, we summarized the characteristics and properties of CREKA peptide, and then described the application research of CREKA-based nanoplatforms in the biomedical field was also in detail. The reported CREKA-based nanoplatforms, including liposome, small extracellular vesicles (sEVs), hybrid nanoparticles, poly (lactic-co-glycolic acid) (PLGA), mesenchymal stell cells, and gelatin, have exhibited excellent performance in cancer imaging, thrombus imaging, antithrombotic therapy, anticancer therapy, bone repair and anti-renal fibrosis (Table 1). Compared with simple small-molecule drugs, nanoparticles based on CREKA exhibited excellent biological tissue-targeting distribution performance, and long cycle time in vivo, improving drug efficacy, and reduced toxic side effects.

**Table 1:** Summary of the application of CREKA-based nanoparticles

Composition of nanoparticles	Characteristics of nanoparticles	Drug loading	Application	References
Ultrasmall manganese ferrite nanoparticles conjugated with CREKA	Acidity, H_2_O_2_ triggered release of Mn^2+^, 4.1 nm–12.4 mV	N.A	Detecting ultrasmall metastasis	[58]
CREKA-targeted gadolinium-based MRI contrast agent	Molecular weight: 2913 Da	N.A	Imaging of micrometastasis	[37]
Poly(lactic-co-glycolic acid) conjugated with CREKA and loading Fe_3_O_4_, IR 780 and ketotifen fumarate	0.457 ± 0.209 mV, 268.87 ± 14 nm,	Fe loading efficiency = 5.74%	Imaging fibrin-rich thrombi and preventing thrombus formation	[64]
Gelatin modified with CREKA and loading MR imaging agent Fe_3_O_4_ (TNPs) and then engulfed by macrophages (MTNPs)	− 6.61 ± 0.76 mV, 197.53 ± 0.83 nm	Fe_3_O_4_ loading rate = 57.81 ± 2.4%	Targeting thrombi imaging by magnet-guided and LIFU responsiveness	[65]
Prussian blue and perfluorinated pentane in the core, CREKA was attached to PLGA as the shell	287.58 ± 6.6 nm, 1 ± 1.35 mV	PB loading efficiency = 35.2%	Achieving antithrombotic therapy by regulating the thrombosis microenvironment	[73]
CREKA-modified mesenchymal stem cells	N.A	The density of CREKA on MSCs was about 9.1*10^11^ molecules per cell	For the repair of myocardial injury	[74]
CREKA-modified polymer nanoparticles and loaded with α-mangostin	− 27.8 ± 0.95 mV, 106.93 ± 3.69 nm	The drug loading of α-mangostin was 49.62%	Lowering the tumor stromal barrier and enhancing the delivery of chemotherapy drugs	[82]
PEGylated liposomes modified with CREKA and loaded doxorubicin	− 28.9 mV, 160 nm	DOX entrapment efficiency was nearly 99%, the modified CREKA was approximately 277.9 ± 13.3 μg/μmol lipids	Enhancing antitumor and anti-metastasis therapy	[83]
IR 780 conjugated with Gd-DOTA and then coassembly with DSPE-PEG-CREKA and loading ticagrelor	− 34.74 ± 5.87 mV, 127.8 ± 12.3 nm	Tic loading rate = 16.7%, entrapment efficiency = 80%	Targeting NIR fluorescence/MRI dual-modal imaging-guided PTT/chemotherapy of tumor	[84]
Iron oxide (Fe-Ms) or manganese oxide (Mn-Ms) core modified with CREKA peptide	20–30 nm r_1_ = 0.170 mM^−1^S^−1^, r_2_ = 456.5 mM^−1^S^−1^	N.A	Non-invasive MR imaging of thrombosis during the pathogenesis of atherosclerosis	[88]
CREKA-modified small extracellular vesicles	90.06 nm	particle-to-protein ratio of CREKA-sEVs was 5 × 10^9^ particles/μg	Targeting therapy for bone repair	[93]
CREKA coupled liposomes and loading celastrol	110 nm, PDI < 0.20	CEL entrapment efficiency > 90%, 57 ± 1.3 μg of CREKA per mg of lipids	Targeted delivery of celastrol to renal interstitial myofibroblasts	[40]

Nevertheless, to achieve the wide application and clinical translation of pharmaceutical preparations based on CREKA in biomedicine, it is necessary to solve some current problems. First, the preparation process of pharmaceutical preparations based on CREKA peptide should be simple, convenient, and easy to prepare; second, CREKA peptide is well suited for nanoparticle targeting modifications because it is small, easy to synthesize, and nearly non-immunogenic. However, CREKA-modified nanoparticles might cause some adverse effects to vary degrees in the long run. So, one should fully understand the in vivo biocompatibility, metabolic pathways, and long-term toxicity of various CREKA-modified nanomaterials. In addition, the targeting properties of the prepared CREKA-modified preparations to diseased tissues should be fully understood, and the CREKA-mediated drug targeting distribution effect needs to be quantified. In summary, CREKA-based drug delivery systems have great potential for the diagnosis and treatment of a wide range of diseases.

## Data Availability

Not applicable.
